# P-214. Primary Prophylaxis for *Clostridioides difficile* Infection in Lung Transplant Recipients: A Retrospective Study

**DOI:** 10.1093/ofid/ofae631.418

**Published:** 2025-01-29

**Authors:** Mark Irwin, Nicole Leedy, Sravanthi Nandavaram, Jaime Soria, Thein Myint

**Affiliations:** University of Kentucky, Lexington, Kentucky; University of Kentucky, Lexington, Kentucky; Massachusetts General Hospital, Boston, Massachusetts; University of Kentucky, Lexington, Kentucky; University of Kentucky, Lexington, Kentucky

## Abstract

**Background:**

There is a paucity of data supporting primary prophylaxis for *Clostridioides difficile* infection (CDI) in lung transplant recipients (LTRs). We aimed to determine if the incidence of CDI is reduced in LTRs receiving primary prophylaxis.

Table 1

**Figure:**
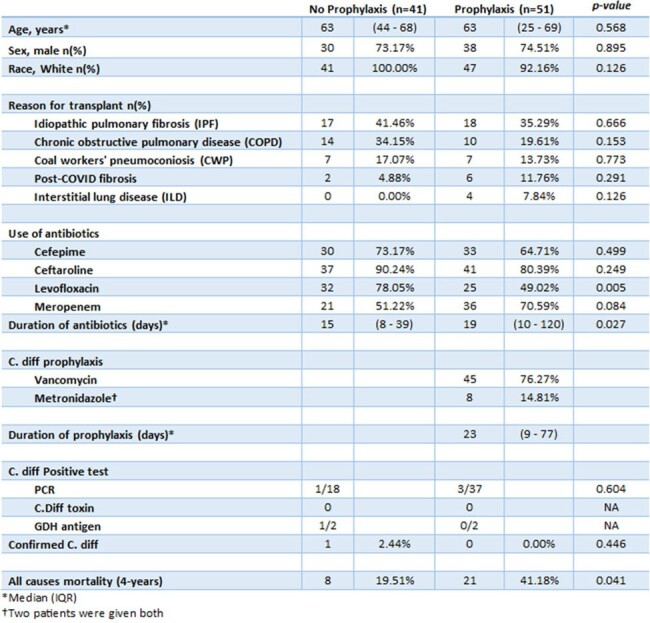

Comparison of clinical characteristics of lung transplant recipients between the two groups.

**Methods:**

A retrospective chart review of all lung transplants from January 2020 to May 2023 was conducted at our institution. CDI was defined as diarrhea with a positive PCR test for the toxin B gene. Primary prophylaxis for CDI at the time of lung transplantation was defined as any use of either oral vancomycin or oral metronidazole in an LTR who received broad-spectrum antibiotics without any history of prior CDI. The incidence of CDI in LTRs was compared between the group with primary prophylaxis and the group without prophylaxis.

**Results:**

Of 92 eligible LTRs, 41 (44.6%) did not receive primary prophylaxis, and 51 (55.4%) received either oral vancomycin (n=45) or oral metronidazole (n=8). The median duration of primary prophylaxis was 23 days (Table1). The median duration of systemic antibiotics was 19 days in the prophylaxis group and 15 days in the no prophylaxis group (p=0.027). *Clostridioides difficile* testing was done in 55 (60.4%) patients within one year after lung transplantation. Three patients had CDI within three months, and one patient had CDI at eight months after lung transplantation.

The incidence of CDI within one year after lung transplantation was 8.1% (3/37) in the prophylaxis group compared to 5.6% (1/18) in the no prophylaxis group based on the *Clostridioides difficile* testing (p=0.60). The incidence would be lower if the LTRs who were not tested for *Clostridioides difficile* due to a lack of concern were included with those who tested negative. All-cause mortality was higher in the prophylaxis group compared to the no prophylaxis group (41.2% vs 19.5%; p=0.041).

**Conclusion:**

Fifty-five percent of LTRs (51/92) received primary CDI prophylaxis, mainly oral vancomycin, at the time of lung transplantation. LTRs who required a longer duration of broad-spectrum antibiotics were more likely to receive primary prophylaxis. There was no difference in the incidence of CDI regardless of primary prophylaxis in LTRs. Our data do not support the use of primary prophylaxis in LTR to reduce incidence of CDI.

**Disclosures:**

**All Authors**: No reported disclosures

